# Dual-Facing Digital Health Systems to Support Self-Management of Chronic Pain: Protocol for a Scoping Review

**DOI:** 10.2196/84152

**Published:** 2026-06-03

**Authors:** Martin Pursley, Fiona Muirhead, Alexandra Mavroeidi, Roma Maguire

**Affiliations:** 1 Physical Activity and Health/Computer Information Sciences, Faculty of Humanities and Social Sciences, University of Strathclyde, 6 Richmond St, Glasgow, G1 1XQ, United Kingdom, 44 07903314961; 2 Physical Activity and Health, Faculty of Humanities and Social Sciences, University of Strathclyde, Glasgow, United Kingdom; 3 Department of Allied Health Professions, Glasgow Caledonian University, Glasgow, United Kingdom; 4 Computer Information Science/Strathclyde Institute Of Pharmacy And Biomedical Sciences, Faculty of Science, University of Strathclyde, Glasgow, United Kingdom

**Keywords:** chronic pain, digital health, pain management, eHealth, self-management

## Abstract

**Background:**

Chronic pain affects approximately 1 in 5 adults worldwide and imposes a major personal and societal burden. Supported self-management, increasingly delivered through digital health systems, is recommended to improve quality of life and function. Dual-facing digital health systems (DDHSs), which engage both patients and professionals, show promise but remain underexplored, with limited use of established frameworks to guide their design and evaluation.

**Objective:**

This protocol outlines the planned methods for conducting a scoping review that aims to map existing DDHSs for chronic pain self-management; examine the frameworks used to inform their design, implementation, and evaluation; and identify gaps to guide future research and practice.

**Methods:**

The review will follow the Joanna Briggs Institute methodology and PRISMA-ScR (Preferred Reporting Items for Systematic Reviews and Meta-Analyses Extension for Scoping Reviews) reporting guidelines. Peer-reviewed studies (2010-present) on DDHSs for use by health care professionals and adults with chronic pain will be included. Searches will be conducted across MEDLINE, CINAHL, Embase, Scopus, Web of Science, and APA PsycInfo, supplemented by citation tracking. Two reviewers will independently screen and extract data using a structured template. Results will be synthesized narratively, with emphasis on system features, populations, and applied frameworks. The quality of the included studies will be assessed using the Quality Assessment for Diverse Studies tool to support the interpretation of the findings.

**Results:**

Searches identified 1377 records, with 822 (59.7%) screened after deduplication. Following full-text assessment, 19 studies were included. The scoping review is expected to be completed by June 2026. The first search was conducted in September 2025, and screening was completed in November 2025. The results are expected to be published in June to August 2026. The evidence base is anticipated to be heterogeneous, with variation in system features, populations, and outcomes. Early indications suggest inconsistent use of theoretical and implementation frameworks, limited integration of dual-facing components, and a predominance of feasibility and acceptability outcomes.

**Conclusions:**

This review will provide a structured overview of DDHSs for chronic pain self-management and the frameworks informing their design and evaluation, with findings intended to guide the development and implementation of future digital interventions.

## Introduction

### Background and Rationale

#### The Problem

Chronic pain is defined by the International Association for the Study of Pain as “an unpleasant sensory and emotional experience associated with, or resembling that associated with, actual or potential tissue damage, persisting for more than 3 months” [1]. It is multifactorial, with biological, psychological, and social factors contributing to the pain experience, and is often associated with significant emotional distress and functional disability [1,2]. Chronic pain is often categorized as “chronic primary pain” or “chronic secondary pain.” Chronic primary pain is “pain in one or more anatomical regions characterized by significant emotional distress (anxiety, anger/frustration, or depressed mood) or functional disability (interference in daily life activities and reduced participation in social roles)” and is an appropriate diagnosis regardless of the observed biological or psychological contributors unless another diagnosis would better explain the symptoms. Chronic secondary pain is chronic pain that exists where an underlying condition (such as osteoarthritis) adequately accounts for the pain experience or impact [2]. It is possible for both to coexist simultaneously within an individual.

#### Prevalence

Chronic pain is a global challenge with significant economic and social burden reported to affect 1 in 5 adults worldwide, although reporting of this is highly variable [3]. Estimates across countries vary, with reported prevalence ranging from 18% in low-income countries [4] to 33% in low- to middle-income countries [5]. Across Europe, prevalence is estimated at 20% [6]. The economic impact of chronic pain has been widely reported. European countries and the United States have consistently reported historical national health care and socioeconomic costs ranging into the billions annually [7-10].

#### The Proposed Approach

Although a cure for chronic pain is not likely in many cases, appropriate interventions can substantially improve quality of life, mood, and functional ability [11]. The Scottish Intercollegiate Guidelines Network and the National Institute for Health and Care Excellence recommend supported self-management approaches in their guidelines for chronic pain. Supported self-management in chronic pain refers to a collaborative approach that enables individuals to manage the physical, emotional, and social impacts of pain with the guidance, encouragement, and support of health care professionals (HCPs) [12]. It differs from self-care in that it involves structured support, often through health care systems, rather than being completely patient led [13].

There is currently no universally accepted definition of self-management interventions (SMIs). Generally, they are understood as structured programs or strategies aimed at improving health outcomes and quality of life by enhancing an individual’s self-efficacy and capacity to manage their condition. In the context of chronic pain, SMIs typically focus on developing skills such as pacing, problem-solving, goal setting, and the use of cognitive and behavioral strategies to regulate symptoms, reduce disability, and foster sustained engagement in valued activities [14,15].

SMIs for chronic pain are increasingly delivered through digital modalities—such as apps, websites, and other eHealth tools—because these formats can overcome common barriers (eg, geographical distance, mobility limitations, and lengthy wait times) that restrict participation in traditional face-to-face programs [16-18]. Digital delivery can take several forms, including web-based programs offering structured psychoeducation and skill training (eg, pacing, goal setting, and cognitive and behavioral strategies); mobile health apps that support symptom tracking, self-monitoring, action planning, and “just-in-time” prompts; telehealth or videoconferencing for synchronous clinician- or peer-led sessions; blended or hybrid models combining digital resources with periodic clinician contact to reinforce skills and accountability; wearable and sensor-based technologies that provide feedback on activity, sleep, and adherence; and moderated digital peer support communities that foster social learning and persistence with behavior change [19].

#### Justification for This Scoping Review

This review will focus on blended or hybrid models of dual-facing digital health systems (DDHSs)—platforms that provide tailored interfaces for both patients and HCPs, enabling reciprocal communication, shared data access, and integrated care management. DDHSs offer a promising approach to support chronic pain management while potentially improving the efficiency of clinical care delivery. However, evidence regarding digitally delivered health interventions remains heterogeneous, encompassing diverse intervention types, populations, and settings, with limited reporting on the application of established frameworks to inform their design, implementation, and evaluation [20]. The development of digital SMIs for chronic pain benefits greatly from the use of established theoretical and methodological frameworks. Guidance such as the UK Medical Research Council’s framework for complex interventions [21]; the Behavior Change Wheel and capability, opportunity, and motivation–behavior model [22]; and the chronic disease self-management model by Holman and Lorig [23] provides structured approaches that help clarify the aims, components, and mechanisms of action of interventions. Applying such frameworks ensures that interventions are not only theoretically informed but also explicitly linked to outcomes that matter to patients, such as symptom regulation, function, and quality of life [2]. Within the field of chronic pain, they also help embed the principles of the biopsychosocial model into digital design, supporting interventions that go beyond symptom reduction to address behavior change and daily functioning. Using recognized frameworks can improve transparency and reporting standards, enable meaningful comparisons across studies, and strengthen the scalability of interventions into clinical pathways. Conversely, neglecting such frameworks risks producing interventions that are poorly specified, inconsistently reported, and challenging to evaluate for effectiveness or cost-effectiveness.

To date, no reviews have specifically examined digital health systems that integrate both patient- and professional-facing components to support chronic pain self-management. Previous reviews have examined related but distinct areas, including digital health technologies designed primarily for HCPs and digital interventions focusing on patients’ experiences of using digital tools for pain management. While these reviews provide valuable insights into each perspective independently, they do not address systems designed to support reciprocal interaction between patients and HCPs, shared access to data, and coordinated self-management support. DDHSs represent a distinct model of digital health intervention in which patient- and clinician-facing components are intentionally integrated to facilitate communication, shared decision-making, and ongoing self-management support. Therefore, a scoping review is needed to systematically map the existing literature on DDHSs for chronic pain, identify the characteristics of these systems, and highlight gaps to inform future research and evidence synthesis. A scoping review is particularly suited due to the potential heterogeneity of the available evidence in the context of the rapidly evolving nature of digital health research [24,25].

### Research Question and Objectives

Accordingly, this scoping review seeks to systematically map the existing evidence on DDHSs that support chronic pain self-management. The review will focus on identifying the characteristics of these systems and the frameworks used to inform their design, implementation, and evaluation to describe the current state of the evidence and highlight gaps in the literature. Therefore, the primary research question is as follows: what DDHSs designed to support self-management of chronic pain currently exist?

The objectives of the review are to (1) characterize existing DDHSs designed to support chronic pain self-management, including user features, types of data collected, outcome measures reported, security and privacy considerations, target populations, and contexts in which the systems are used (eg, primary or secondary care); (2) identify and describe frameworks or models that have been used to guide the design, implementation, or evaluation of DDHSs for supported self-management of chronic pain; (3) examine how these frameworks are reported and applied within intervention development and evaluation; and (4) identify gaps in the use and reporting of frameworks within the existing literature to inform future research and development of DDHSs for chronic pain self-management.

## Methods

The proposed scoping review will be conducted in accordance with the Joanna Briggs Institute (JBI) methodology for scoping reviews [26] and will be reported according to the PRISMA-ScR (Preferred Reporting Items for Systematic Reviews and Meta-Analyses Extension for Scoping Reviews) guidelines [27]. This protocol was developed prior to completion of the review; sections have been updated where necessary to reflect methodological steps that have since been undertaken.

### Eligibility

#### Overview

The inclusion and exclusion criteria were defined using the population, concept, and context framework outlined in the JBI methodology for scoping reviews. A more detailed overview of the eligibility criteria can be found in Textbox 1.

Textbox 1. **Description of the eligibility criteria based on the population, concept, and context framework for study inclusion in this scoping review.**

**Inclusion criteria**

- Adults (≥18 y) with chronic pain (≥3-mo duration) supported by a health care professional (HCP) directly involved in their care
- HCPs, including nurses, pharmacists, general practitioners (GPs), physiotherapists, occupational therapists, psychologists, social care professionals, and related professionals involved in chronic pain management
- Dual-facing digital health systems (eg, eHealth, mobile health, telehealth, and web-based interventions) designed to support chronic pain self-management
- Systems that enable reciprocal communication, shared data access, and integrated care between patients and HCPs
- Systems that include tools supporting information exchange, such as symptom tracking, outcome scoring, monitoring, shared decision-making, goal setting, education, exercise advice, and self-care interventions (eg, cognitive behavioral therapy, acceptance and commitment therapy, mindfulness, and meditation)
- Systems that have both patient-facing and clinician-facing interfaces
- Systems targeting chronic pain specifically (≥3-mo duration), including chronic primary pain (eg, fibromyalgia, complex regional pain syndrome, and chronic primary headache) and chronic secondary pain (eg, osteoarthritis and cancer-related pain)
- Studies conducted in any research, clinical, or community setting where HCPs manage patients with chronic pain (eg, primary, secondary, or tertiary care; pharmacies; GP practices; hospitals; residential facilities; and patients’ homes)
- No restrictions on study design

**Exclusion criteria**

- Patients under 18 y
- Digital health systems that do not include both patient- and professional-facing components
- Systems not designed to support chronic pain self-management
- Systems focused solely on acute or nonchronic pain
- Systems without contextual relevance to chronic pain self-management
- Non–peer-reviewed sources (eg, editorials, opinion pieces, and magazine or newspaper articles)
- Studies not written in English
- Systematic, scoping, or narrative reviews (individual studies from such reviews may be screened if relevant)
- Study protocols (eg, trial registries)

#### Population

Studies exploring DDHSs for collaborative use between an HCP and a patient will be included. The intended end users for these systems must be HCPs and their associated patients (aged 18 years or older) with chronic pain. The HCP must be directly involved in the associated patient’s care. “HCP” refers to any professional who provides a health and care service, including but not limited to nurses, pharmacists, general practitioners, physiotherapists, occupational therapists, psychologists, and social care professionals. There will be no restrictions on the type of health and care profession, and there will be no restrictions on the source or type of chronic pain experienced by the patients.

#### Concept

The concept of interest for this review will be DDHSs (including but not limited to eHealth, mobile health, telehealth, and web-based interventions) intended to support people in the self-management of chronic pain. Any chronic pain–specific digital health systems that are designed to enable reciprocal communication, shared data access, and integrated management of care between patients and HCPs will be considered in this review. This may include systems intended for direct exchange of information (eg, symptom tracking, outcome measure scoring, activity monitoring, and sleep diaries), shared decision-making, goal setting, education, exercise advice, and self-care interventions (eg, low-level cognitive behavioral therapy, acceptance and commitment therapy, mindfulness, and meditation). Solutions may include mobile apps, web-based applications, or any other tools provided digitally, but they must have both a patient-facing and a clinician-facing interface.

Digital health solutions for the management of all types of chronic pain conditions will be considered, including chronic primary pain (eg, fibromyalgia, complex regional pain syndrome, and chronic primary headache), chronic secondary pain (eg, osteoarthritis and chronic pain secondary to cancer), or both [2,11]. Digital health solutions must be chronic pain specific, that is, for the management of a diagnosed chronic pain condition, with pain defined as “chronic” if it lasts 3 or more months.

#### Context

There will be no restrictions on the context in this review. Contexts may include research and clinical settings, that is, any environment in which HCPs may be involved in the management of patients with chronic pain. Settings will likely include primary, secondary, and tertiary care settings (eg, pharmacies, general practitioners, and hospitals) and community settings (eg, residential facilities and the patients’ homes).

### Inclusion Criteria

Studies exploring DDHSs intended to support people in the self-management of chronic pain via collaborative use between an HCP and an adult patient will be included. Chronic pain is defined according to the *International Classification of Diseases, 11th Revision*, as persistent or recurrent pain lasting 3 or more months. Where studies refer to “chronic pain” without specifying a duration, we will assume alignment with the *International Classification of Diseases, 11th Revision*, definition; however, studies explicitly describing pain of less than 3 months, sometimes also referred to as “chronic pain,” will be excluded, reflecting ongoing inconsistency in terminology within the literature.

No restrictions will be placed on the source or type of chronic pain experienced by the patient. Peer-reviewed studies including adult participants (≥18 years), published after 2010, and written in English will be included. Studies including mixed-age populations will be included only in cases in which data related to adult participants can be clearly distinguished; studies in which adult data cannot be separated will be excluded.

### Exclusion Criteria

Studies will be excluded if the systems studied lack both professional- and patient-facing components and do not support chronic pain self-management, defined as enabling patients to interpret information and take action in daily life rather than serving solely as clinician-led monitoring or surveillance, and if the studies focus on acute or nonchronic pain, involve patients under 18 years of age, or are published in non-English languages before 2010.

### Operationalization of DDHS

To support consistent application of the eligibility criteria, DDHSs will be operationalized as systems that include both patient-facing and clinician-facing components with functional integration between the two. At a minimum, included systems must meet the following criteria: (1) a patient-facing component (enables active self-management support, such as symptom tracking, goal setting, access to tailored educational content, or delivery of SMIs [eg, exercise programs and cognitive or behavioral strategies]), (2) a clinician-facing component (enables HCPs to access patient-generated data and/or provide input into patient care [eg, monitoring progress, tailoring recommendations, or communicating with the patient]), and (3) integration between the 2 components (the system must support shared access to data and/or reciprocal interaction between patients and HCPs [eg, bidirectional data flow, feedback, or communication]).

Systems will be excluded if they (1) have only one interface (patient only or clinician only), (2) provide clinician access to data without enabling self-management support, or (3) function solely as passive monitoring or record-keeping tools without integration into self-management processes.

In cases in which eligibility is unclear (eg, systems embedded within electronic health record portals or those with limited clinician interaction, such as asynchronous messaging), inclusion decisions will be based on whether the system demonstrates meaningful integration of patient self-management support with clinician oversight or input rather than on the presence of separate interfaces alone.

### Types of Sources

The types of sources of evidence to be included in this scoping review will be limited to peer-reviewed primary research studies. No restrictions will be placed on study design. Systematic reviews and meta-analyses will be screened to identify additional eligible primary studies but will not be included in the final synthesis.

Gray literature (eg, dissertations, reports, and conference proceedings) will not be included. This decision was made to ensure consistency in reporting quality and prioritize studies that provide sufficient methodological detail regarding system design, implementation, and evaluation to support data charting and reproducible synthesis. While this approach may result in the underrepresentation of commercially developed or nonevaluated digital health systems, it is consistent with the aim of mapping the characteristics of systems described in the academic literature and will be considered when interpreting the findings.

Opinion papers, editorials, and letters will be excluded as they do not provide primary data relevant to the review objectives. Sources will be limited to the English language and will be sought from 2010 onward to reflect contemporary digital health technologies.

### Search Strategy

A comprehensive search strategy was developed in consultation with an academic librarian. The following electronic databases will be searched: MEDLINE, CINAHL, Embase, Scopus, Web of Science, and APA PsycInfo. Subject headings and keywords specific to each database were used to combine synonyms related to the population, concept, and context.

The reference lists of included studies will be hand searched, and backward citation searching will be conducted to identify additional relevant studies. Given the heterogeneity in the terminology used to describe such systems, eligibility will be determined based on functional characteristics rather than reliance on specific labels.

The full search strategies for all databases are provided in Multimedia Appendix 1.

### Data Management and Screening

All references will be imported into EndNote (Clarivate Analytics) for deduplication and then uploaded to Rayyan (Qatar Computing Research Institute) for screening. Prior to full screening, a subset of studies will be independently screened by members of the review team to ensure consistent interpretation and application of the inclusion and exclusion criteria. Discrepancies will be discussed and resolved, and the screening approach will be refined where necessary. Studies that are deemed to be entirely nonrelevant based on their title and abstract (ie, studies where there is no uncertainty about their lack of congruence with the inclusion criteria) will be excluded by the first author (MP). The same process will be simultaneously undertaken by another independent researcher. A consensus on the studies to be included for full-text screening after this step will be reached through discussion, and any disagreements will be resolved by a third researcher. The lead author will then undertake full-text screening of the remaining articles, whereas a sample of 20% of these articles will be reviewed in full independently by a second researcher, as described by Petticrew and Roberts [28], to ensure that the eligibility criteria are appropriately applied. Backward citation screening will be conducted iteratively by MP on a source paper basis. This will involve manually reviewing the reference lists of the included studies, with references screened at the title and abstract level to identify potentially relevant studies, with full texts retrieved in cases in which relevance to the review question is unclear or potentially met. A PRISMA (Preferred Reporting Items for Systematic Reviews and Meta-Analyses) flow diagram will document the process.

### Data Charting (Extraction)

A standardized data charting form was developed using the JBI data extraction instrument for scoping reviews as an initial guide and was subsequently refined for this review. The final charting form captures study identification details, participant characteristics of both patients and HCPs, and detailed information relevant to the review questions. This includes the characteristics of the DDHS, such as its name, data collected digitally, procedures of the intervention, duration or dose where reported, patient-facing and professional-facing components, and the nature of interaction between users and professionals. Additional sections capture self-management domains targeted, theoretical frameworks or models cited and how explicitly these were applied, implementation-related factors, evaluation focus, broadly reported outcomes, and analytic notes relevant to the conceptual contribution and positioning of each study in relation to the DDHS.

The charting process will be undertaken iteratively (Multimedia Appendix 2). The form was piloted independently on an initial sample of 3 included studies by members of the review team, and discrepancies in extraction informed further refinement of both the charting form and an accompanying data charting codebook for sections D to G, which required greater interpretation and categorization. The codebook was developed to support consistency and transparency in extracting intervention characteristics, self-management domains, theoretical content, implementation issues, and evaluation focus. It specifies that data will be extracted at the level of the individual paper only rather than the broader intervention program and that items will be coded positively only in cases in which they are explicitly described in the paper. No inferences will be made from related publications, supplementary papers, or clinical assumptions; where information is absent or unclear, it will be coded as “not reported,” with any nuance recorded in the “notes” column rather than used to upgrade a code.

For system-level characteristics, information will be recorded only in cases in which it is explicitly reported in the included paper. For example, details related to data handling, privacy, security, governance, integration, workflow implications, or sustainability will be charted in cases in which the study provides sufficient description. General or implied statements will not be treated as evidence that a feature is present. This approach was adopted to improve reproducibility and reduce overinterpretation in an area in which reporting is often heterogeneous. The revised charting form is provided in Multimedia Appendix 3, and the accompanying codebook was used to guide subsequent extraction.

Any disagreements in data charting will be resolved through discussion within the review team, with involvement of a fourth reviewer where required.

### Data Synthesis and Presentation

Due to the anticipated heterogeneity of digitally delivered SMIs for chronic pain, a narrative synthesis will be used to summarize and interpret the evidence. Studies will be grouped by characteristics such as intervention components, target population, and framework use. Key findings, patterns, and gaps will be presented in tables and discussed textually, with particular attention to how frameworks inform intervention design, implementation, and evaluation, as well as to implications for future research. Consistent with the aims of a scoping review, a formal synthesis of intervention effectiveness will not be conducted. However, outcomes reported in individual studies will be extracted descriptively to map the types of measures used to evaluate DDHSs.

### Handling of Missing Data

As this is a scoping review, no analysis of effect size will be undertaken. Data charting will focus on mapping key study characteristics and concepts rather than on quantitative synthesis. In cases in which it is not possible to extract one or more data items from the source material, this will be noted explicitly in the data charting form.

Where feasible, corresponding authors will be contacted to request clarification or additional information. If such efforts are unsuccessful, the study will remain eligible for inclusion, and missing data will be reported transparently. In these cases, only the information available in the published record will be used, with gaps clearly indicated to ensure clarity and reproducibility.

### Quality Assessment

Although formal quality assessment is not essential when undertaking a scoping review, it may be useful to support the authors in drawing conclusions from what is likely to be a diverse and heterogeneous body of literature. Therefore, quality assessment will be conducted using the Quality Assessment for Diverse Studies appraisal tool (Multimedia Appendix 4) [29].

### Ethical Considerations

No ethics approval is required as this review involves analysis of publicly available data. Findings will be disseminated through peer-reviewed publications, conference presentations, and stakeholder workshops.

## Results

**Figure 1.**
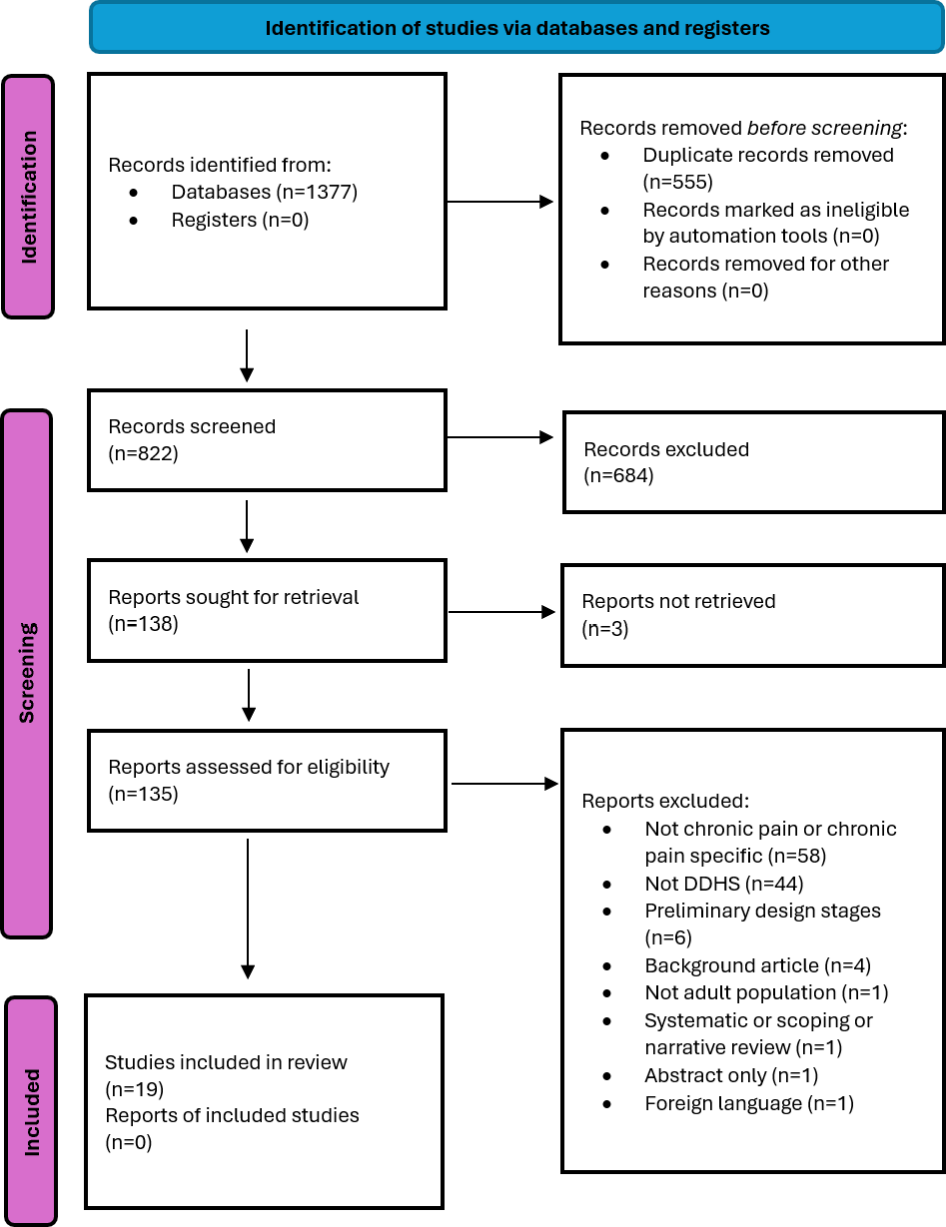
PRISMA (Preferred Reporting Items for Systematic Reviews and Meta-Analyses) flowchart to show the selection of studies. DDHS: dual-facing digital health system.

This study was funded in April 2025 as part of a PhD studentship. Database searches identified 1377 records, of which 822 (59.7%) were screened following deduplication. Following title and abstract screening and full-text assessment, 19 studies met the inclusion criteria (Figure 1). The first search was conducted in September 2025, and screening was completed in November 2025. Data analysis is currently ongoing, and the scoping review is expected to be completed by June 2026, with publication anticipated in June to August 2026.

It is anticipated that the evidence identified will be highly heterogeneous, encompassing a wide range of delivery formats, populations, and outcome measures, with limited standardization across studies. The review is also expected to reveal inconsistent use and reporting of established design, implementation, and evaluation frameworks. In addition, comparatively few interventions may fully integrate both patient- and professional-facing components, indicating a gap in the development of more holistic systems. The outcomes assessed in the included studies are anticipated to focus primarily on feasibility, acceptability, or user satisfaction rather than robust measures of effectiveness or long-term impact. Overall, the findings are expected to demonstrate a rapidly evolving but uneven evidence base, highlighting important opportunities to guide future research and inform the design, implementation, and evaluation of digital health interventions for chronic pain self-management.

## Discussion

### Overview

Despite the growing adoption of digital health interventions for chronic pain management, recent reviews highlight significant gaps in understanding the optimal implementation strategies and the role of theoretical frameworks in guiding intervention design and evaluation [16,20,30]. To our knowledge, no studies to date have reviewed dual-facing (patient and HCP) digital health systems that support the self-management of chronic pain.

This scoping review aims to provide a comprehensive foundation for future research on digital health solutions that support chronic pain care through systems combining both patient- and professional-facing components. By systematically mapping the available systems, including their core components, target populations, delivery settings, and implementation and evaluation processes, this review will clarify current practices and inform the future design of more effective, user-centered interventions. Furthermore, by examining the reporting and use of implementation and evaluation frameworks, the review will identify gaps in methodological rigor and provide guidance for structuring future interventions to enhance usability, fidelity, and integration into routine clinical workflows. While this scoping review will not assess the overall effectiveness of these combined digital systems, it will synthesize outcomes and features reported in individual studies, offering actionable insights to support the design, implementation, and systematic evaluation of future patient- and professional-facing interventions for chronic pain.

### Strengths and Limitations

A key strength of this scoping review will be its systematic and rigorous methodology. The review will follow the JBI framework for scoping reviews and adhere to the PRISMA-ScR reporting guidelines, ensuring transparency, reproducibility, and methodological rigor. An expert academic librarian collaborated in the development of comprehensive and tailored search strategies. Search terms were constructed using database-specific subject headings (eg, MeSH [Medical Subject Headings] for MEDLINE) alongside relevant keywords identified from previous, related reviews to maximize coverage of the literature. Together, these procedures support a robust and reproducible approach to identifying and synthesizing the available evidence.

This scoping review will have some limitations that should be acknowledged. First, the heterogeneity of digital SMIs for chronic pain—including variation in delivery modalities, components, target populations, and outcomes—may limit the extent to which findings can be synthesized in a structured or comparative manner.

Second, the review is restricted to English-language publications. While translation of non–English-language studies is feasible, this decision was made to ensure consistency and accuracy in data extraction, particularly given the level of detail required to characterize DDHSs, including intervention components, theoretical frameworks, and implementation features. Accurate interpretation of these elements often requires a nuanced understanding of terminology and context, which may not be reliably captured through automated or limited translation approaches. However, this restriction may result in the exclusion of relevant studies published in other languages.

In addition, the review is limited to peer-reviewed literature, and therefore, commercially developed, service-level, or nonevaluated digital health systems not reported in the academic literature may be underrepresented. While this approach was adopted to ensure consistency and sufficient methodological detail for data charting, it may limit the capture of real-world implementations.

Third, the review focuses exclusively on adult populations (≥18 years) and, therefore, excludes studies involving pediatric or adolescent populations, as well as mixed-age samples where adult data cannot be clearly distinguished. This decision was made to maintain conceptual and clinical consistency as self-management approaches, health care pathways, and digital intervention design may differ between younger and adult populations; however, it may limit the generalizability of findings across the full age spectrum of individuals with chronic pain.

Fourth, the review relies on studies that explicitly report the use of design, implementation, or evaluation frameworks; inconsistent or incomplete reporting may constrain the ability to fully assess how frameworks are applied across interventions. Finally, the rapidly evolving nature of digital health technologies means that new interventions or approaches may emerge shortly after the review is completed, potentially limiting the temporal relevance of the findings.

This protocol highlights the need for a scoping review to identify combined professional- and patient-facing digital health solutions that support self-management of chronic pain and to explore the implementation and evaluation frameworks used in their design and delivery. The results of this review will highlight the available digital health solutions that support the self-management of chronic pain and will provide information regarding the systems’ components, the contexts in which they are used, and their implementation and evaluation methods. This scoping review will be conducted using a systematic and transparent approach to maximize the inclusion of relevant literature. Nonetheless, it is acknowledged that certain digital health interventions may be omitted, particularly those developed through commercial channels without accompanying academic dissemination or those published in languages other than English. The findings will contribute to the evolving evidence base in digital health and offer insights into the potential utility of digital technologies to enhance supported self-management of chronic pain.

## Supplementary material

10.2196/84152 Multimedia Appendix 1 Draft example of MEDLINE (Ovid) search strategy.

10.2196/84152 Multimedia Appendix 2 Data charting codebook (sections D-G).

10.2196/84152 Multimedia Appendix 3 Blank data charting template.

10.2196/84152 Multimedia Appendix 4 Quality Assessment for Diverse Studies tool (adapted from Harrison et al [29]).

10.2196/84152 Checklist 1 PRISMA-ScR checklist.

## Abbreviations

DDHS: dual-facing digital health system
HCP: health care professional
JBI: Joanna Briggs Institute
MeSH: Medical Subject Headings
PRISMA: Preferred Reporting Items for Systematic Reviews and Meta-Analyses
PRISMA-ScR: Preferred Reporting Items for Systematic Reviews and Meta-Analyses Extension for Scoping Reviews
SMI: self-management intervention
