# Supplementary material for: Dual-Facing Digital Health Systems to Support Self-Management of Chronic Pain: Protocol for a Scoping Review
Source: JMIR Res Protoc. 2026 Jun 3;15:e84152. doi: 10.2196/84152 (PMC13232601; doi:10.2196/84152)

# Appendix B – Draft example of MEDLINE (Ovid) search strategy

1. ("chronic pain") or ("persist* pain" or "pain persist*") or ("long term pain")

2. (“musculoskeletal pain” or “MSK pain”) or (“joint pain”) or (“multiple joint pain” or “widespread pain” or “whole body pain” or “diffuse pain”) or (“osteoarthritis” or “arthritis” or “arthritic pain”) or (“rheumatoid arthritis” or “rheumatic*”) or (“psoriatic arthritis” or “psoriatic arthropathy”) or (“axial spondyloarthritis” or “ankylosing spondylitis”) or (“polymyalgia rheumatica” or “PMR”) or (“giant cell arteritis” or “GCA” or “temporal arteritis”) or (“systemic lupus erythematosus” or “lupus” or “SLE”) or (“back pain” or “low back pain”) or (“radiculopathy” or “radicular pain” or “sciatica” or “nerve root pain”) or (“spinal stenosis”) or (“knee pain” or “patellofemoral pain” or “PFPS” or “anterior knee pain”) or (“foot pain” or “heel pain” or “plantar fasciitis”) or (“ankle pain”) or (“hip pain” or “greater trochanter pain” or “greater trochanteric pain syndrome” or “GTPS”) or (“hand pain” or “thumb pain”) or (“wrist pain”) or (“elbow pain” or “tennis elbow” or “golfers elbow”) or (“carpal tunnel syndrome” or “cubital tunnel syndrome”) or (“shoulder pain” or “rotator cuff related shoulder pain” or “RCRSP” or “subacromial pain syndrome” or “SAPS” or “shoulder impingement”) or (“frozen shoulder” or “capsulitis” or “adhesive capsulitis”) or (“neck pain” or “cervical pain” or “cervical radiculopathy”) or (“thoracic pain”) or (“tendon pain” or “tendinopathy” or “tendonitis” or “tendinosis”) or (“fibromyalgia” or “fibromyalgia syndrome” or “FMS”) or (“complex regional pain syndrome” or “CRPS”) or (“chronic headache” or “chronic migraine” or “cervicogenic headache”) or ("chronic fatigue syndrome" or "chronic fatigue" or "myalgic encephalomyelitis" or “ME”) or ("irritable bowel syndrome" or "irritable bowel" or “IBS”) or (“endometriosis”) or (“testicle pain” or “testicular pain”)

3. self ADJ3 manag* (MEDLINE Ovid)

4. ("digital app" or "digital application") or (“digital intervention” or “digital treatment”) or (“digital tool”) or ("mobile app" or "mobile application") or ("apps") or ("smartphone" or "smartphone app" or "smartphone application") or (“telehealth" or “ehealth" or "mhealth") OR "digital health" OR “ehealth” OR “health app” OR “digital platform*" OR "online platform*" OR "patient portal*" OR "electronic health record*" OR EHR OR "clinical decision support system*" OR “CDSS” OR "health information technolog*" OR “HIT”

5. "patient-facing" OR "patient centred" OR "patient centered" OR "patient portal*" OR "patient interface*" OR "patient app*" OR "patient dashboard*" OR "self-management" OR "self monitor*" OR "consumer health information"

6. "clinician-facing" OR "provider-facing" OR "professional-facing" OR "healthcare provider*" OR "health professional*" OR "clinician dashboard*" OR "clinical interface*" OR "decision support" OR "shared platform" OR “bidirectional” OR "dual-facing" OR "two sided"

7. 1 OR 2

8. 7 AND 3

9. 8 AND 4

10. 9 AND (5 AND 6)

11. 10

12. limit 11 to (english language and humans and yr="2010 -Current")


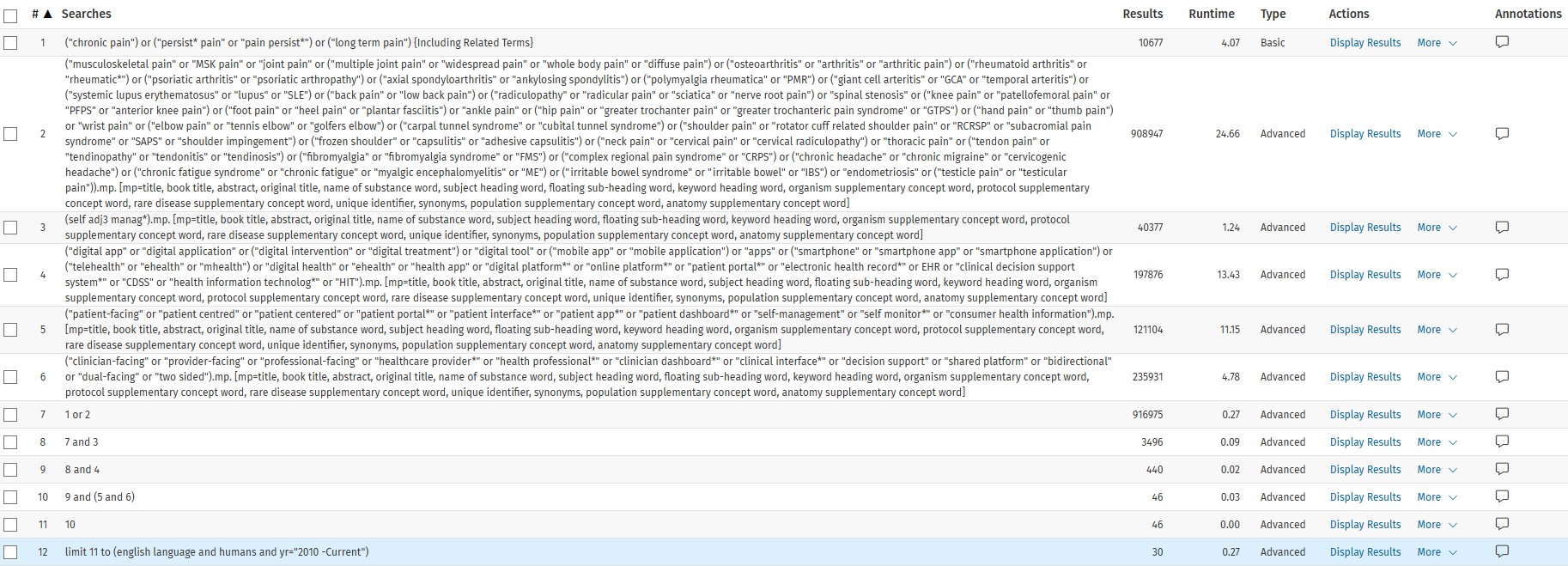

Supplement: Multimedia Appendix 1 [file resprot-v15-e84152-s001.docx]
