# Supplementary material for: Dual-Facing Digital Health Systems to Support Self-Management of Chronic Pain: Protocol for a Scoping Review
Source: JMIR Res Protoc. 2026 Jun 3;15:e84152. doi: 10.2196/84152 (PMC13232601; doi:10.2196/84152)
Supplement: Multimedia Appendix 2 [file resprot-v15-e84152-s002.docx]

# Data Charting Codebook (Sections D-G)

This codebook was produced following undertaking the initial data extraction sample of Achten, Al Sharaa and Andrews by MP, HM and AM. This was created in response to discrepancies in data extraction using the charting table for sections D through G. These sections require interpretation, categorisation, and judgement therefore some discrepancies were expected. This codebook was developed to guide subsequent data extraction.

## General Principles (Apply to All Sections)

1. Code at the paper level, not the broader programme (extract based only on what this specific paper reports, not what is known from related publications i.e. RCTs, development papers, multi-paper series using same DDHS)
2. Only code YES if explicitly described in this paper.
3. Do not infer from:
   1. Related RCTs
   2. Supplementary publications
   3. Clinical common sense
4. If unclear, code NO and note “not explicitly reported”.
5. Use Notes column for nuance, not for upgrading a NO to a YES.

| **D: DDHS Intervention** | |
| --- | --- |
| General details | |
| Name of DDHS | Record exact name used in the paper. |
| Data collected by DDHS | List only data explicitly described as collected digitally.  Do not infer data types unless clearly stated. |
| Procedures of intervention | Describe what the paper says the system does.  Avoid interpretation beyond reported content. |
| Number of times intervention was delivered/what period of time (N sessions, schedule, duration, intensity or dose) | Record only if explicitly stated.  If duration known but session frequency unclear, state exactly that. |
| Patient-facing components (Code YES only if the feature is explicitly described as part of the digital system) | |
| Education | YES if:  Educational content delivered digitally (text, video, modules)   NO if:  Education occurs only face-to-face |
| Symptom/activity tracking | YES if:  Patients enter symptom, pain, or activity data into the system   NO if:  Symptoms discussed but not digitally recorded |
| Exercise/tasks | YES if:  Exercises are delivered, prescribed, or monitored via the digital system   NO if:  Exercises given face-to-face only |
| Messaging/feedback | YES if:  Direct digital communication (e.g., in-app messaging, automated feedback)   NO if:  Feedback only during in-person sessions |
| Other (specify) |  |
|  |  |
| Professional-facing components | |
| Dashboard/monitoring | YES if:  Clinicians can view patient data via dashboard or digital interface   NO if:  No clinician-facing interface described |
| Messaging | YES if:  Clinicians send or receive messages through system   NO if:  No explicit digital messaging functionality |
| Decision support | YES only if:  - Automated prompts  - Algorithms  - Alerts  - Structured recommendations embedded in system   NO if:  Clinicians interpret raw data themselves |
| Care coordination | YES only if:  System supports coordination across providers, services, or transitions   NO if:  System simply embedded within a single service |
| Other (specify) |  |
|  |  |
| Nature of interaction | |
| App/device used |  |
| One-way monitoring | YES if:  Data flows from patient to clinician  No digital response mechanism |
| Two-way interaction | YES only if:  Digital communication occurs both directions within system |
| Shared decision-making elements | YES only if:  The digital interface itself supports joint decision processes   No if:  SDM occurs only in face-to-face sessions |
| **E: Self-management & theory** | |
| Self-management domains targeted (Code YES only if explicitly targeted, measured, or described as a goal) | |
| Symptom regulation | YES if:  Strategies explicitly intended to help patients manage or modulate symptoms directly are included (e.g. Pacing strategies targeting pain flares, Relaxation techniques, Breathing strategies, Cognitive coping tools, Pain management skills training, Flare-up planning, Behavioural strategies explicitly aimed at symptom control)  NO if:  Symptom change is indirect consequence of exercise/education |
| Activity / function | YES if:  Intervention aims to improve activity or function explicitly |
| Psychological processes | YES only if:  Constructs (e.g., acceptance, self-efficacy, fear) explicitly targeted or measured   NO if:  Psychological benefit implied but not operationalised |
| Education / knowledge | YES if:  Explicit educational component about condition/self-management |
| Healthcare navigation | YES only if:  System supports navigating services (e.g. getting the most out of your appointment), referrals, care pathways etc. |
|  |  |
| Frameworks or models cited (Code YES only if explicitly named. Mentioning behaviour change without naming a framework = NO) | |
| COM-B / Behaviour Change Wheel | Code YES only if explicitly named. Mentioning behaviour change without naming a framework = NO |
| ACT | Code YES only if explicitly named. Mentioning behaviour change without naming a framework = NO |
| Theoretical Domains Framework (TDF) | Code YES only if explicitly named. Mentioning behaviour change without naming a framework = NO |
| Self-Determination Theory | Code YES only if explicitly named. Mentioning behaviour change without naming a framework = NO |
| Other (specify) | Code YES only if explicitly named. Mentioning behaviour change without naming a framework = NO |
| None reported |  |
|  |  |
| How explicitly applied | |
| Design | YES if:  Framework informed system development |
| Evaluation | YES if:  Framework used to analyse outcomes |
| Mentioned only | YES if:  Named but not applied |
| Not clear | YES if:  Theoretical assumptions implied but not explicitly stated |
|  |  |
| **F: Implementation focus** | |
| Framework named e.g. CFIR, RE-AIM, NASSS, Normalisation Process Theory... (Specify in notes column) | YES only if:  Implementation framework explicitly named (CFIR, RE-AIM, NASSS etc.) |
| Implementation strategies described (Specify in notes column) | YES only if:  Discrete strategies described (training, workflow redesign, champions, audit)  Embedding into routine care = NO. |
| Barriers to implementation | Record only if barriers explicitly analysed or discussed. |
| Facilitators for implementation | Record only if facilitators explicitly analysed or discussed. |
| Sustainability/scale considerations | YES only if:  Long-term embedding, scale-up, or system integration discussed explicitly  Short duration alone = NO. |
|  |  |
| **G: Evaluation focus** | |
| Primary evaluation focus (Code based on the stated study aim — not the intervention type) | |
| Feasibility | YES only if:  Study explicitly framed as feasibility/pilot |
| Usability | YES only if:  Usability testing conducted or explicitly analysed |
| Acceptability | YES only if:  Acceptability measured or central analytic focus |
| Engagement | YES only if:  Usage metrics analysed (logins, adherence, completion) |
| Clinical outcomes | YES only if:  Study powered/designed to assess clinical change  Exploratory symptom tracking = NO. |
| Service use / cost | YES only if:  Economic or utilisation outcomes measured |
| Implementation | YES only if:  Implementation outcomes systematically evaluated |
|  |  |
| Outcomes broadly reported | |
| Pain | Code YES only if measured using explicit instruments or quantitative metrics.  Qualitative discussion ≠ outcome measurement. |
| Function | Code YES only if measured using explicit instruments or quantitative metrics.  Qualitative discussion ≠ outcome measurement. |
| Quality of life | Code YES only if measured using explicit instruments or quantitative metrics.  Qualitative discussion ≠ outcome measurement. |
| Psychological outcomes | Code YES only if measured using explicit instruments or quantitative metrics.  Qualitative discussion ≠ outcome measurement. |
| Utilisation (e.g. healthcare service use or costs as an outcome) | Code YES only if measured using explicit instruments or quantitative metrics.  Qualitative discussion ≠ outcome measurement. |

## Domain E Definitions

Self-management domains were categorised using an analytic framework derived from established chronic disease self-management and behaviour change literature. The domains were informed by conceptual models that distinguish medical or symptom management, role or functional management, and emotional management as core components of self-management (Lorig & Holman, 2003), alongside contemporary behaviour change theory emphasising capability, opportunity, and motivation as determinants of behaviour (Michie et al., 2011). These domains were further informed by models commonly applied in chronic pain rehabilitation, which emphasise activity engagement, psychological flexibility, and patient education as central treatment components (Nicholas et al., 2011). Together, these perspectives provided a structured yet flexible conceptual basis for mapping intervention targets across heterogeneous dual-facing digital health systems.

The identified domains were applied as analytic categories to facilitate structured comparison across interventions, consistent with methodological guidance for scoping reviews (Arksey & O’Malley, 2005; Peters et al., 2020). The review did not seek to test or privilege a single theoretical framework; rather, domains were used pragmatically to map how included studies conceptualised and operationalised self-management within digital systems. Where theoretical frameworks were explicitly cited within individual studies, these were extracted separately to preserve distinctions between review-level analytic categorisation and author-reported theory use.

### Symptom regulation

Intervention components explicitly intended to help individuals monitor, modulate, or cope with symptoms (e.g., pain flares, fatigue), including structured coping strategies, pacing techniques, or symptom-management skills. Symptom change occurring indirectly through exercise or education alone does not constitute symptom regulation.

### Activity / function

Intervention elements designed to improve physical activity levels, functional ability, or participation in daily roles. This includes graded exercise, activity pacing for functional restoration, or behavioural activation aimed at increasing engagement in meaningful activities.

### Psychological processes

Explicit targeting or measurement of cognitive, emotional, or motivational constructs relevant to self-management (e.g., self-efficacy, fear avoidance, psychological flexibility, pain acceptance). Psychological benefit implied but not operationalised through defined constructs does not meet this criterion.

### Education/knowledge

Provision of structured informational content intended to improve understanding of the condition, treatment rationale, or self-management strategies (e.g., pain neuroscience education, condition-specific guidance).

### Healthcare navigation

Intervention components that support patients in navigating healthcare systems, accessing services, coordinating care pathways, or making informed decisions about service use. Routine embedding within a single service without explicit navigation support does not qualify.
